# Functional processing and secretion of Chikungunya virus E1 and E2 glycoproteins in insect cells

**DOI:** 10.1186/1743-422X-8-353

**Published:** 2011-07-15

**Authors:** Stefan W Metz, Corinne Geertsema, Byron E Martina, Paulina Andrade, Jacco G Heldens, Monique M van Oers, Rob W Goldbach, Just M Vlak, Gorben P Pijlman

**Affiliations:** 1 Laboratory of Virology, Wageningen University, Droevendaalsesteeg 1, 6708 PB Wageningen, The Netherlands; 2 Erasmus MC, Department of Virology, 3000 CA Rotterdam, The Netherlands; 3 Nobilon International BV, Boxmeer, The Netherlands

## Abstract

**Background:**

Chikungunya virus (CHIKV) is a mosquito-borne, arthrogenic *Alphavirus *that causes large epidemics in Africa, South-East Asia and India. Recently, CHIKV has been transmitted to humans in Southern Europe by invading and now established Asian tiger mosquitoes. To study the processing of envelope proteins E1 and E2 and to develop a CHIKV subunit vaccine, C-terminally his-tagged E1 and E2 envelope glycoproteins were produced at high levels in insect cells with baculovirus vectors using their native signal peptides located in CHIKV 6K and E3, respectively.

**Results:**

Expression in the presence of either tunicamycin or furin inhibitor showed that a substantial portion of recombinant intracellular E1 and precursor E3E2 was glycosylated, but that a smaller fraction of E3E2 was processed by furin into mature E3 and E2. Deletion of the C-terminal transmembrane domains of E1 and E2 enabled secretion of furin-cleaved, fully processed E1 and E2 subunits, which could then be efficiently purified from cell culture fluid via metal affinity chromatography. Confocal laser scanning microscopy on living baculovirus-infected *Sf*21 cells revealed that full-length E1 and E2 translocated to the plasma membrane, suggesting similar posttranslational processing of E1 and E2, as in a natural CHIKV infection. Baculovirus-directed expression of E1 displayed fusogenic activity as concluded from syncytia formation. CHIKV-E2 was able to induce neutralizing antibodies in rabbits.

**Conclusions:**

Chikungunya virus glycoproteins could be functionally expressed at high levels in insect cells and are properly glycosylated and cleaved by furin. The ability of purified, secreted CHIKV-E2 to induce neutralizing antibodies in rabbits underscores the potential use of E2 in a subunit vaccine to prevent CHIKV infections.

## Background

Chikungunya virus (CHIKV) is an arthropod-borne (arbo)virus that causes epidemics in Africa, India and South-East Asia [1]. Recent outbreaks in Italy in 2007 [2] and autochthonous transmission events in France in 2010 [3] exemplify the threat of continued spread of CHIKV in the Western world, which correlates with the concurrent expanding distribution of its insect vector. CHIKV is maintained in a sylvatic transmission cycle of mosquitoes, rodents and primates, with *Aedes aegyti *as the primary vector. However, the responsible vector causing the severe CHIKV epidemic on the Reunion Islands in 2005/2006 was *Ae. albopictus *[4]. This vector switch made the virus endemic in more temperate regions and resulted in the first European cases (Italy, 2007) of transmission by local populations of *Ae. albopictus *[1,5].

CHIKV (family *Togaviridae*: genus *Alphavirus*) has a single-stranded positive-sense RNA genome, which varies slightly in length between different isolates, but is approximately 11,800 nts long [6] and encodes two open reading frames (ORF). The RNA is encapsidated in a nucleocapsid of approximately 40 nm in diameter [7]. The nucleocapsid is tightly enveloped by a host-derived lipid bilayer (envelope) supporting the virus-encoded envelope proteins. Eighty glycoprotein spikes are C-terminally anchored within the viral envelope and are exposed on the surface of virions and of infected cells.

The nonstructural proteins required for viral RNA replication are directly translated from the 5' two-thirds region of the viral genome. The structural polyprotein is translated from a viral subgenomic mRNA (sgRNA), located at the 3'one-third part of the genome [8,9]. The five structural proteins (capsid, E3, E2, 6K, E1) are translated as a single polyprotein, from which capsid (C) is autocatalytically cleaved off to encapsidate new plus-strand RNA molecules. The envelope polyprotein precursor E3-E2-6K-E1 is then translocated to the endoplasmatic reticulum (ER). Host signalases process the polyprotein at the N- and C-terminal end of the 6K peptide, resulting in E3E2 (also known as precursor E2: PE2), 6K and E1 [10], all anchored in the ER membrane. After this proteolytic cleavage, E3E2 and E1 will eventually form heterotrimers in the early Golgi compartment. Subsequently, E3E2 undergoes a furin-dependent maturation cleavage in the trans-Golgi system at the consensus cleavage signal R-X-(K/R)-R. This furin cleavage is not a prerequisite for virion assembly [11]. The hetero-trimeric spikes consisting of E2 and E1 facilitate cell receptor recognition, cell entry via pH-dependent endocytosis and support viral budding [9].

The major clinical symptoms of a CHIKV infection are febrile illness and severe joint pains [12]. Recently, macrophages were identified as being key players in CHIKV infection, persistence and pathogenesis. Macrophage-derived pro-inflammatory products are strongly involved in the muscle and joint immunopathological findings after alphavirus infection [13,14]. Currently, there are no specific treatments for CHIKV infections and no licensed vaccine for any alphavirus is available for human use. During an infection with the alphavirus type species Sindbis virus (SINV) neutralizing antibodies are generally directed against E2 and to a lesser extent to E1. This holds true for other alphaviruses as well, suggesting that E1 and E2 are conserved among alphaviruses as epitope donors [15,16]. Therefore glycoproteins E1 and E2 serve as the major targets in the development of a (subunit) vaccine against CHIKV infections. A recently developed, experimental CHIKV vaccine based on virus-like particles (VLPs) containing both E1 and E2 induced a protective immune response in non-human primates [17]. While this VLP approach may be a way forward in the development of a CHIKV vaccine, the described method of transfecting large DNA plasmids into mammalian cells remains challenging in terms of upscaling. Therefore, a subunit approach may be better compatible with industrial operations [18,19].

In this study, the baculovirus-insect cell expression system was used to study recombinant CHIKV glycoprotein subunit formation. This production system is a safe and efficient way of expressing heterologous proteins on a large scale in eukaryotic cells [20] and the proven technology has resulted in several commercially available veterinary vaccines [21], including a veterinary subunit vaccine targeting Classical Swine Fever [18] and a human vaccine against cervical cancer (Cervarix, GlaxoSmithKline) [22]. A recombinant human vaccine against influenza virus (FluBlok) Biological License Application is currently in the process of evaluation by the Food and Drug Administration in the USA [19]. Recombinant protein expression by baculoviruses is based on the use of the strong polyhedrin promoter and the exchange of the polyhedrin gene for the heterologous gene of interest. This late phase baculoviral protein is expressed in large amounts in infected cells, but is not essential for baculovirus replication [23]. Furthermore, protein expression in insect cells allows post-translational modifications, accurate folding and efficient secretion [21,24]. Drosophila cells have recently been used to efficiently express and process CHIKV-glycoprotein complexes [25], yet there are few reports on successful expression of alphavirus structural polyproteins with recombinant baculoviruses [26,27], and there are no studies that describe the expression of individual full-length alphavirus E1 and/or E2 glycoproteins. Here, we investigated the expression of individual CHIKV E1 and E2 glycoproteins in insect cells and analyzed the routing and post-translation modifications of these glycoproteins as well as the fusogenic properties of recombinant E1. In addition, purification methods were developed for secreted forms of these glycoproteins, of which the ability to generate a neutralizing antibody response was investigated.

## Results

### Expression of CHIKV-E2 and -E1 glycoproteins in *Sf*21 insect cells by recombinant baculoviruses

**Figure 1:**
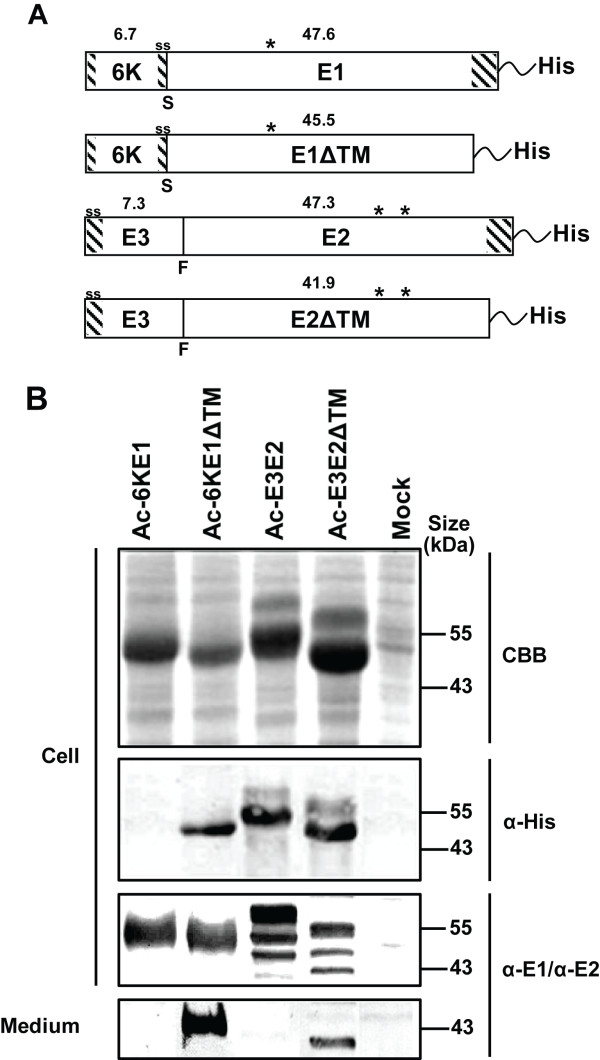
**CHIKV E1 and E2 expression using recombinant baculoviruses**. A) Schematic representation of four CHIKV glycoprotein constructs expressed from recombinant baculoviruses. The shaded areas represent transmembrane domains or signal peptides (ss), asterisks indicate predicted N-glycosylation sites, S and F indicate signalase and furin cleavage sites, respectively. The predicted molecular mass of the glycoproteins is indicated. All constructs were equipped with a C-terminal 6 × His-tail. B) Gene expression in the cell and in medium fractions of infected Sf21 cells was analyzed by Coomassie brilliant blue-staining and western blotting using α-His mabs and rabbit α-E1 and rabbit α-E2 polyclonal antisera.

Four recombinant baculoviruses were generated to express membrane attached and secreted versions of CHIKV glycoproteins E1 and E2 (Ac-6KE1, Ac-6KE1ΔTM, Ac-E3E2 and Ac-E3E2ΔTM) (Figure 1A). The glycoprotein coding sequences were cloned downstream of the polyhedrin promoter in an *Autographa californica *multicapsid nucleopolyhedrovirus (AcMNPV) bacmid from which the promoters and open reading frames of the p10, cathepsin and chitinase genes were deleted (See Materials and Methods). All four glycoprotein constructs were equipped with a C-terminal 6 × his tag. *Sf*21 insect cells were infected with the respective recombinant baculoviruses at a MOI of 10 TCID_50 _units per cell and cells were harvested 72 h post infection (hpi), which appeared to be the optimal time point for harvesting (data not shown). Recombinant protein content in the cell fraction was analyzed by Coomassie brilliant blue (CBB)-staining and western analysis using α-His monoclonal antibodies (mabs) and α-E1 and α-E2 polyclonal antisera (Figure 1B).

CBB staining of *Sf*21 cell lysates showed that CHIKV-E2 and -E1 were expressed at very high levels. The size of the proteins observed after infection with Ac-6KE1 and Ac-6KE1ΔTM (Figure 1B, lanes 1 and 2) closely matched the predicted molecular mass of 6KE1 and 6KE1ΔTM of 54.2 kDa and 52.1 kDa, respectively. CHIKV 6KE1 was not detected by western analysis using α-His mabs, but was recognized well by α-E1 polyclonal antiserum. This is most likely caused by the proteolysis of the C-terminal his-tag of CHIKV 6KE1. Expression analysis of insect cells infected with Ac-E3E2 and Ac-E3E2ΔTM (Figure 1B, lane 3 and 4) resulted in two polypeptides for each construct. The smallest ones correspond to the predicted molecular mass of unprocessed E3E2 (54.6 kDa) and E3E2ΔTM (49.2 kDa), respectively. The larger ones, estimated to be 3-4 kDa larger in size, are possibly glycosylated forms of E3E2 and E3E2ΔTM, respectively. When the cell fractions were analyzed with the α-E2 polyclonal antiserum, a third, smaller protein was seen, which most likely represents furin-processed, glycosylated E2 and E2ΔTM, respectively. To allow secretion of E1 and E2 into the cell culture fluid, the C-terminal transmembrane domains of E1 and E2 were deleted (constructs Ac-6KE1ΔTM and Ac-E3E2ΔTM). Medium fraction analysis shows that the removal of the C-terminal TM of E1 and E2 indeed resulted in secretion of E1 and E2 (Figure 1B, bottom, lanes 2 and 4).

### Glycosylation status of baculovirus expressed CHIKV-E1 and -E2

**Figure 2:**
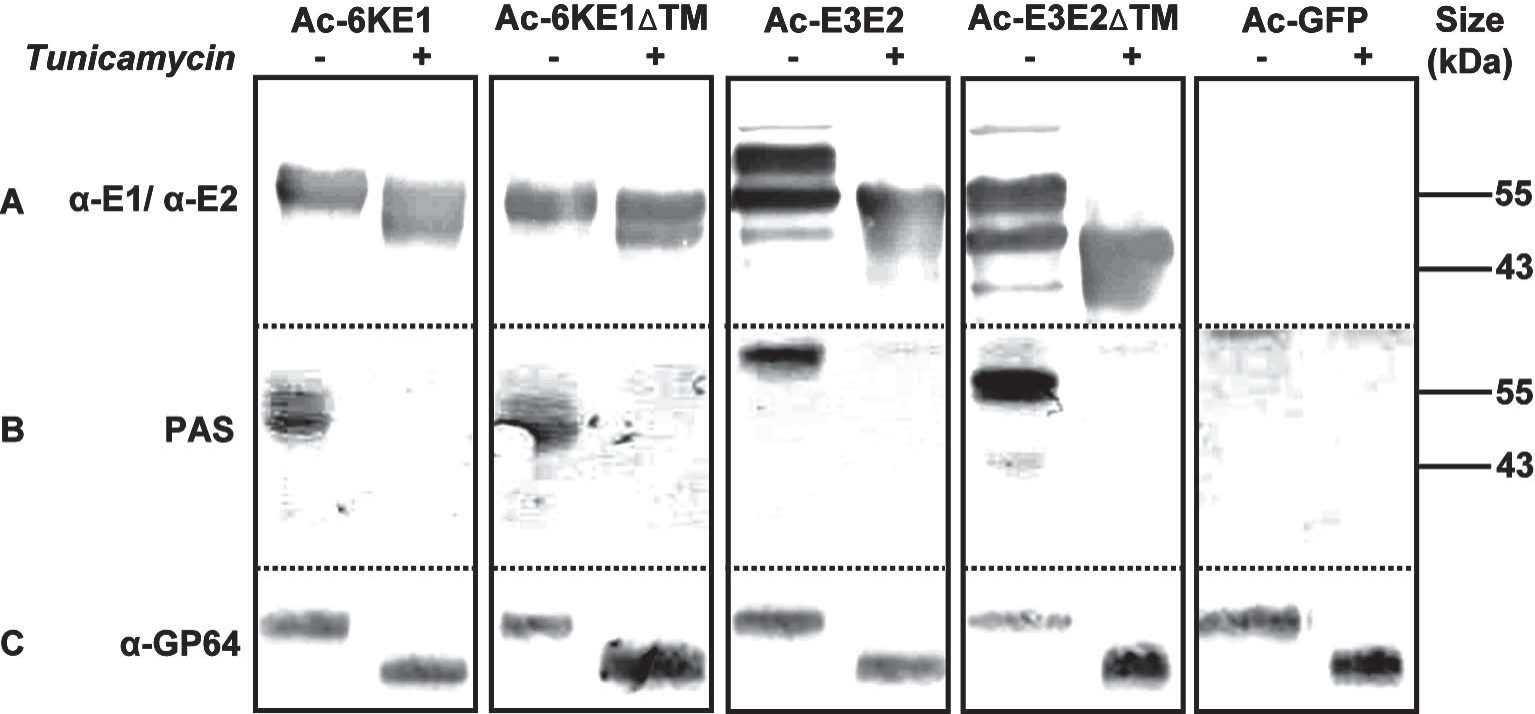
**N-linked glycosylation of CHIKV E1 and E2 synthesized with baculovirus vectors**. A) Infected Sf21 cells were treated with tunicamycin and cell fractions were analyzed by western blotting using α-E1 and α-E2 polyclonal antisera. B) Glycosylation of recombinant CHIKV E1 and E2 was confirmed by PAS-staining. C) Detection of baculovirus glycoprotein GP64 verified tunicamycin activity.

To analyze whether the size difference observed between the upper and lower bands of E3E2 or E3E2ΔTM in CBB and western blots (Figure 1B) can be attributed to glycosylation of E2 a tunicamycin assay was performed to analyze the N-glycosylation status of both glycoproteins. It is common knowledge that alphavirus E1 and E2 are N-linked glycosylated, but the number of glycosylation sites can vary among species [28-31]. CHIKV-E1 is predicted to be glycosylated at N141 and CHIKV-E2 at N263 and N273 [32]. *Sf*21 cells were infected with the 4 recombinant baculoviruses, respectively, (Figure 1A) and incubated in the presence or absence of tunicamycin, harvested at 72 hpi and separated from the medium fraction. Whole cell lysates were analyzed by western blotting (Figure 2A) and by Periodic Acid Schiff staining (Figure 2B), which stains glycosyl groups on proteins [33].

Expression of 6KE1 and 6KE1ΔTM in tunicamycin treated *Sf*21 cells resulted in a band with lower molecular mass (Figure 2A, left). This suggests that E1 is at least partially glycosylated. PAS-staining of 6KE1 and 6KE1ΔTM resulted in protein bands with a size similar to the proteins detected by western blotting and confirmed that CHIKV-E1 was glycosylated. Western blot detection using α-E2 polyclonal antiserum showed that infection with Ac-E3E2 and Ac-E3E2ΔTM resulted in the triple-band pattern previously observed (Figure 1B). The minor fourth upper-band that was detected is only observed occasionally and may represent E2 aggregates. Upon tunicamycin treatment of Ac-E3E2 and Ac-E3E2ΔTM-infected cells, the E2-specific polypeptides with the highest molecular mass (~58 and ~55 kDa, respectively) disappeared, suggesting that these are the N-glycosylated forms of E3E2 and E3E2ΔTM (Figure 2A). PAS-staining, which is absent in the tunicamyin treated lanes, confirmed this result. Thus it can be concluded that the bands of ~55 kDa and ~49 kDa in infections with Ac-E3E2 and Ac-E3E2ΔTM, respectively, represent non-glycosylated E3E2 and E3E2ΔTM proteins. The smaller polypeptides of ~50 kDa and ~44 kDa, correspond in size to glycosylated, furin-cleaved E2 and E2ΔTM, respectively. PAS-staining on the Ac-E3E2ΔTM infected cell fraction also stains this additional protein of ~44 kDa (Figure 2B), suggesting that glycosylated, furin-cleaved E2ΔTM is indeed produced, albeit in relatively low abundance. Immunodetection of the baculovirus GP64 envelope protein was used as a positive control (Figure 2C). GP64 is essential for baculovirus budding and cell entry, and is known to be heavily glycosylated [34,35] at 4 of the 5 predicted positions [36]. As expected, a significant decrease (~6 kDa) in molecular mass was observed as a result of tunicamycin treatment. GP64 is best detected on highly concentrated budded virus preparations and more difficult in insect cells. PAS staining is not sensitive enough to detect glycans on minor amounts of GP64, compared to overexpressed CHIKV glycoproteins, which explains the lack of PAS staining in cells infected the Ac-GFP control virus (Figure 2B, right).

These results show that a substantial fraction of CHIKV E1 and E2 proteins expressed by recombinant baculoviruses in *Sf*21 insect cells is N-glycosylated and that only a minor fraction of the glycosylated E3E2 precursor is processed by furin into mature, N-glycosylated E2.

### Furin processing of recombinant CHIKV-E3E2 and -E3E2ΔTM

During natural alphavirus glycoprotein maturation, E3 is released from E3E2 via cleavage by a furin-like protease [11]. However, the triple-band patterns found during infection with Ac-E3E2 and Ac-E3E2ΔTM (Figure 2A, right) suggest that the bulk of intracellular E3E2 is incompletely processed by furin into the individual E3 and E2 proteins. To investigate the level of furin-processing and to confirm the nature of the smallest polypeptide in the triple-band pattern, *Sf*21 cells were infected with Ac-E3E2 and Ac-E3E2ΔTM in the presence or absence of furin inhibitor.

**Figure 3:**
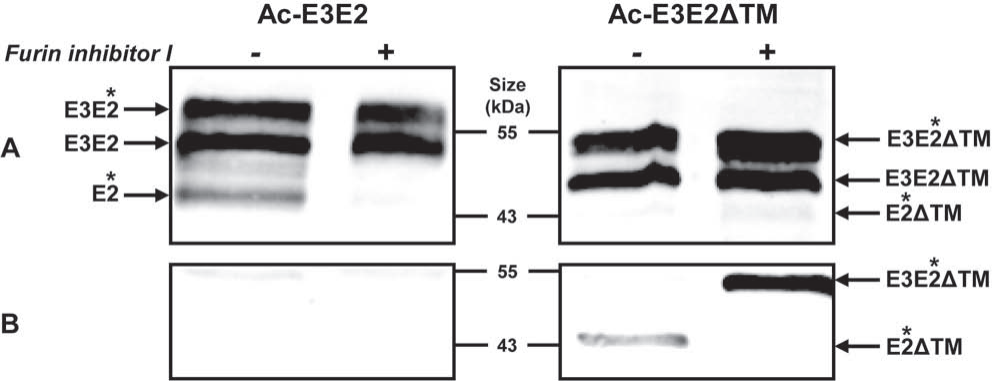
**Furin inhibiton assay on Ac-E3E2 and Ac-E3E2ΔTM infected *Sf*21 cells**. Sf21 cells were infected with Ac-E3E2 and Ac-E3E2ΔTM in the absence (-) or presence (+) of furin inhibitor. The cell fraction (A) and the concentrated medium fraction (B) were analyzed by western blotting using α-E2 polyclonal antiserum. Glycosylated proteins are indicated with an asterisk.

Western blot analysis of Ac-E3E2 infected cells shows that the smallest polypeptide (~44 kDa) detected by anti-E2 antiserum disappeared in the presence of furin inhibitor (Figure 3A, left) indicating that this represents the mature E2, which according to the previous experiment was also N-glycosylated (Figure 2). As expected, all E2-specific polypeptides were retained in the cell fraction (Figure 3B, left) unless the TM was deleted (Figure 3B, right) in accordance with the experiments showing that E2 is only secreted when the C-terminal TM-domain was deleted (Figure 1B). It should be noted that furin-processed E2ΔTM could hardly be observed in the cell fraction, which might suggest that the protein is secreted shortly after it is cleaved by furin in the trans-Golgi complex. Interestingly, when furin cleavage was inhibited in Ac-E3E2ΔTM-infected cells, the N-glycosylated fraction of E3E2ΔTM was still secreted into the culture fluid (Figure 3B, right). It can therefore be concluded that secretion of E2ΔTM is not dependent on furin processing.

### Secretion of recombinant CHIKV-E1ΔTM and -E3E2ΔTM from insect cells

The recombinant baculovirus expression analysis and the furin inhibition assay showed that CHIKV E1ΔTM and E2ΔTM are secreted from *Sf*21 cells when they are expressed with their native signal peptide 6K and E3, respectively (Figures 1B and 3B). To investigate whether other heterologous signal peptides might have a stronger effect on protein secretion, 6K and E3 were both exchanged for the honey bee melittin signal peptide (HBM), which has been shown to improve both expression and secretion of heterologous proteins in baculovirus infected cells [37]. In other constructs, the first 41 amino acids from 6K were deleted as they are not part of the signal peptide necessary for ER translocation of E1. The remaining 6K signal sequence was cloned upstream of E1ΔTM (Ac-6K^-40^E1ΔTM) and of E2ΔTM (Ac-6K^-40^E2ΔTM). The level of intracellular or secreted E1 and E2 was compared by western blotting. A control lane of CHIKV-infected Ap61 mosquito cells was loaded to indicate the size of native E1 and E2 proteins.

**Figure 4:**
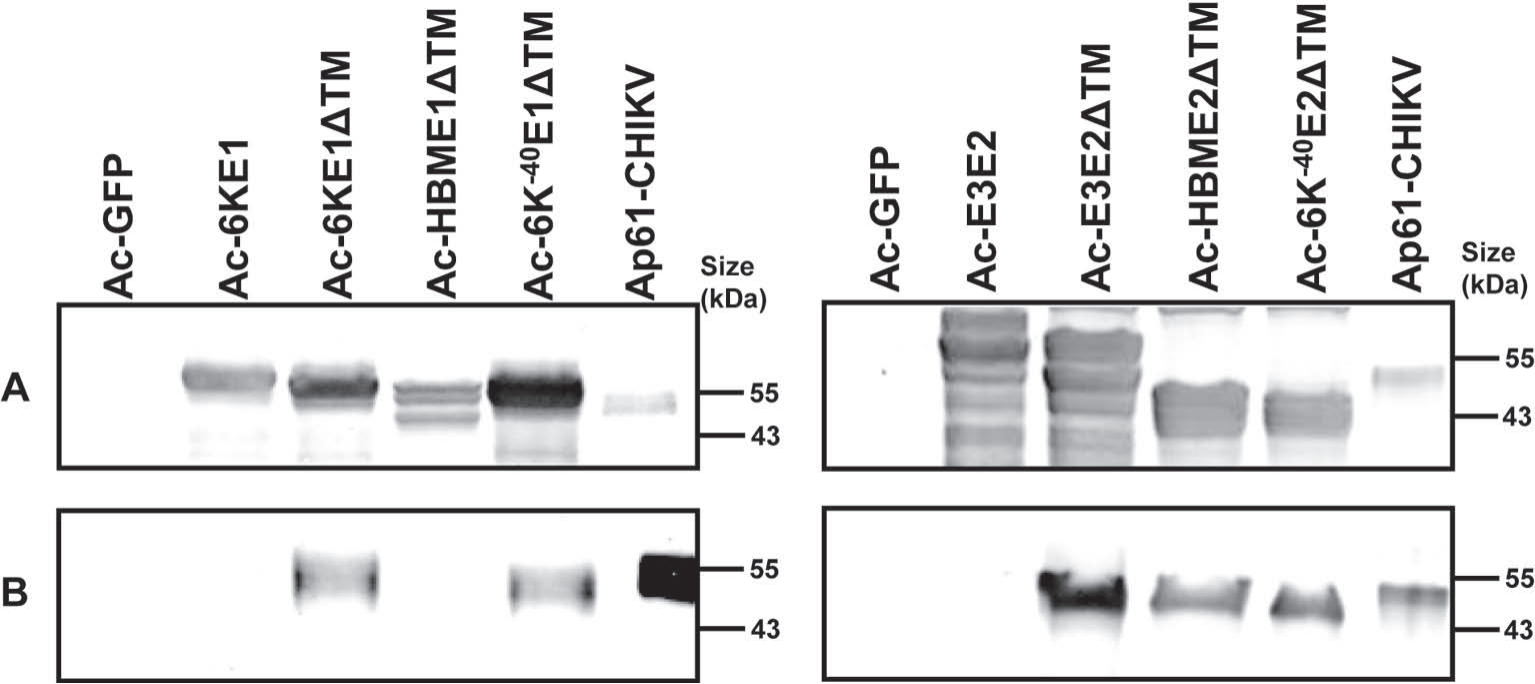
**CHIKV E1ΔTM and E2 protein secretion**. Both CHIKV E1ΔTM and E2ΔTM were expressed with E3, HBM or 6K^-40 ^as signal sequence. The cell fraction A) and the medium fraction B) were analyzed by western blot using α-E1 and α-E2 polyclonal antibodies. CHIKV infected *Ap*61 mosquito cell-lysate was used as a control.

The replacement of 6K for HBM upstream of E1ΔTM resulted in lower intracellular E1 levels (Figure 4A), while the secretion level was also dramatically decreased (Figure 4B). Deletion of the first 41 amino acids from 6K resulted in equal secretion levels compared to the native complete signal peptide 6K. It could be concluded that the signal sequence in 6K is sufficient for efficient secretion of E1ΔTM. When the same signal peptides were exchanged with E3 to test whether E2ΔTM secretion could be enhanced, all constructs were expressed successfully (Figure 4A), but medium fraction analysis showed that neither the HBM nor the 6K^-40 ^signal sequence could improve E2ΔTM protein secretion. This indicates that the CHIKV-6K and E3 signal peptides direct E1ΔTM and E2ΔTM secretion better than other well studied signal peptides like the HBM signal sequence. These results suggest that the CHIKV signal peptides could potentially serve as strong signal peptides for secretion of other heterologous proteins.

### Purification of secreted recombinant CHIKV-E1ΔTM and -E2ΔTM

**Figure 5:**
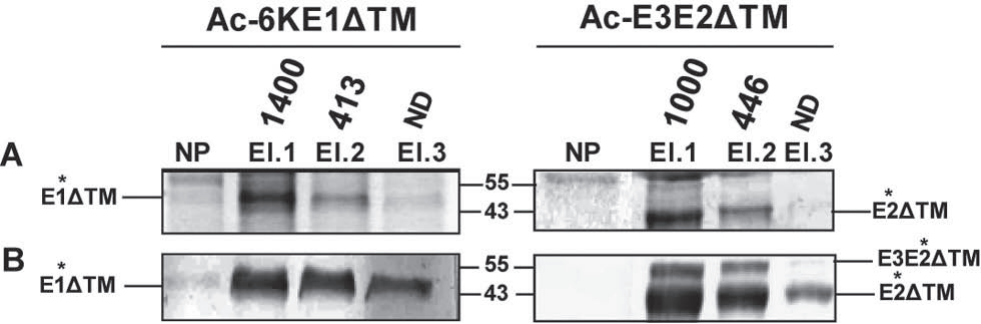
**Purification of secreted, recombinant CHIKV E1ΔTM and E2ΔTM proteins**. *Sf*21 cells were infected with Ac-6KE1ΔTM and Ac-E3E2ΔTM and the respective serum-free medium fractions were loaded onto Talon^® ^spin columns. Bound proteins were eluted 3 times with 150 mM imidazol. The elution fractions were analyzed with CBB (A) and western blotting, using α-E1 and α-E2 polyclonal antisera (B). NP represents non-purified fractions and El.1-3 indicate the subsequent elution fractions. Total protein concentration of the elution fractions (1 ml each) are noted above in μg/ml.

Both Ac-6KE1ΔTM and Ac-E3E2ΔTM constructs were tagged with a C-terminal polyhistidine-tail for detection and purification purposes. Medium fraction analyses showed that E1ΔTM and E2ΔTM were effectively secreted from the cell, which enables protein purification by Co^2+^-histidine interaction. Total medium fractions were loaded onto Talon^® ^spin columns and bound proteins were eluted with 150 mM imidazol. CBB analyses of the three elution fractions (Figure 5A) showed a dramatic increase in concentration of both E1ΔTM and E2ΔTM compared to the non-purified fractions, indicating that both recombinant proteins were efficiently purified from the medium fraction. Western analyses using the polyclonal antisera (Figure 5B) confirmed that the purified fractions indeed contained E1ΔTM and E2ΔTM and that multiple elution steps are required to completely elute the recombinant CHIKV glycoproteins from the column. Two bands were detected in the elution fractions of Ac-E3E2ΔTM (Figure 5B, right). The protein band with the highest molecular mass most likely represents glycosylated E3E2ΔTM, which complies with the finding that secretion of E2ΔTM is not dependent on furin cleavage (Figure 3B). Protein concentrations in the elution fractions were determined by Bradford assay and resulted in a total yield of 38 and 30 mg/l secreted protein for CHIKV-E1ΔTM and CHIKV (E3)E2ΔTM, respectively, at a cell concentration of 6.7 × 10^5 ^Sf21 cells/ml. Metal affinity purification of secreted CHIKV-E1ΔTM and -E2ΔTM resulted in a protein recovery of ~60% and >95%, respectively.

### Surface expression of CHIKV-E1 and -E2 in *Sf*21 cells

Alphavirus glycoproteins are, during a natural infection, expressed at the surface of the host cell to allow budding of progeny virus [7]. From the foregoing experiments it became clear that baculovirus expressed CHIKV E1 and E2 are (at least partially) glycosylated and secreted from *Sf*21 cells when their C-terminal TM domain is deleted. It was also observed that E3E2 is (at least partially) processed by furin, suggesting that the posttranslational processing of CHIKV glycoproteins in baculovirus-infected cells may resemble the processing during a natural CHIKV infection [10]. If this is the case, E1 and E2 are expected to be transported to the cell membrane during baculovirus infection and be exposed at the cell surface.

**Figure 6:**
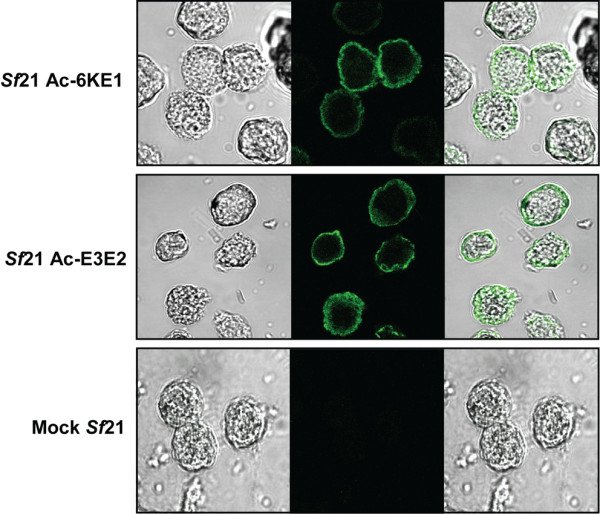
**Surface expression of CHIKV E1 and E2 on the plasma membrane**. *Sf*21 cells were infected with recombinant baculoviruses and living cells were immunostained against E1 and E2. Confocal microscopy reveals the presence of CHIKV-E1 and-E2 at the surface of infected *Sf*21 cells.

Therefore, non-permeable, living *Sf*21 cells infected with Ac-6KE1 or Ac-E3E2 were subjected to immunofluorescence using polyclonal E1 and E2 antisera [38]. Positive staining indicated that the glycoproteins are exposed at the surface of the cell (Figure 6). From the ring-like structure observed by confocal microscopy (Figure 6, top and middle) it could be concluded that both E1 and E2 are translocated to the plasma membrane when expressed in insect cells. This ring-like structure was not observed in mock infected cells (Figure 6, bottom).

### Fusogenic activity of CHIKV-E1 expressed in *Sf*21 cells

**Figure 7:**
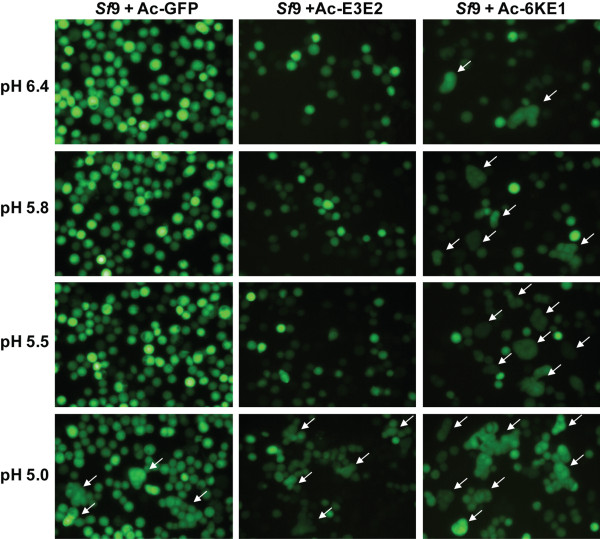
**Syncytia formation after surface expression of CHIKV-E1 in insect cells**. *Sf*9-ET cells in cholesterol supplemented Sf900 II medium (pH = 6.4) were infected with Ac-6KE1, Ac-E3E2 and Ac-GFP. Cells were incubated 72 h post infection with medium of pH = 5.8, pH = 5.5 and pH = 5.0 for 2 min. Pictures were taken 4 h post induction. Syncytia are indicated with arrows.

This study has shown that production of CHIKV E1 and E2 insect cells using baculovirus vectors leads to glycosylation, (partial) furin processing and plasma membrane translocation and/or secretion of E1 and E2. The individually expressed glycoproteins appear to follow similar processing steps as compared to wildtype CHIKV infections [10], which might imply that the recombinant glycoproteins retained their functionality. The following experiment was designed to test the fusogenic activity of baculovirus expressed E1, which in CHIKV infection regulates fusion during endocytosis in a pH-dependent manner, with a pH-optimum of 5.5 [39]. Since alphavrus fusion is cholesterol dependent [40] and insect are cholesterol auxotrophs, *Sf*9-ET cells were cultured in cholesterol-supplemented Sf900 II insect medium (pH = 6.4) and were infected with Ac-6KE1, Ac-E3E2 and Ac-GFP. *Sf*9-ET is a transgenic cell line expresing eGFP under the control of the polyhedrin promoter, thereby only allowing eGFP expression during baculovirus replication [41]. In this way, baculovirus infected cells could be easily visualized. Therefore, *Sf*9-ET cells infected with Ac-GFP express higher amounts of GFP, compared to Ac-6KE1 and Ac-E3E2 infected *Sf*9-ET cells. Cells were treated with acidified medium of pH = 5.8, pH = 5.5 and pH = 5.0. Cells were screened for syncytia formation 4 h post treatment. Syncytia formation after induction with medium of pH6.4, pH = 5.8 or pH = 5.5 was only observed in cells producing CHIKV-E1 at 72 h.p.i (Figure 7, right). In contrast, cells infected with Ac-E3E2 (Figure 7, middle) or Ac-GFP (Figure 7, left) did not form syncytia, unless they were induced with acidified medium of pH = 5.0 (Figure 7, bottom). At this low pH, baculovirus GP64 induces syncytia formation [42]. Thus, expression of E1 is correlated with syncytium formation and E1 is functionally active as viral fusion protein in a defined pH range. The fusogenicity of CHIKV E1 is in the same order of magnitude to what has been found for the major envelope fusion protein F of baculoviruses with this particular assay suggesting that E1 is highly fusogenic [43]. These data concerning pH-dependent syncytia formation exclude the possibility that the syncytia formation in this assay at pH values of 5.8 and 5.5 was caused by the baculovirus infection itself. These findings are in line with the surface expression of CHIKV-E1 (Figure 6) and indicate that E1 retains its fusogenic activity when expressed in *Sf*-cells.

### Virus neutralization test

Purified CHIKV-E1ΔTM and -E2ΔTM (Figure 5) were used to generate polyclonal antisera in rabbits. To analyse the neutralizing activity of the polyclonal α-E1 and α-E2, the antisera were incubated with 100 TCID_50_/ml CHIKV and subsequently incubated for 4 days with BHK-21 cells. Total suppression of cytopathic effect was used as a microscopic marker for virus neutralization. Rabbits vaccinated with CHIKV-E2ΔTM developed neutralizing antibody titers against CHIKV, up to a dilution of 1:40. No neutralizing antibodies were measured in sera of the animals vaccinated with CHIKV-E1ΔTM. The sera of CHIKV infected mice was used as a control and neutralized CHIKV up to 1:320 dilution. These results show that purified CHIKV-E2ΔTM, expressed by recombinant baculoviruses, is able to induce neutralizing antibodies in rabbits.

## Discussion

The continued spread of CHIKV worldwide, the associated threat for invasion of Europe and other Western countries, and the economic impact of a CHIKV epidemic highlights the demand for an effective antiviral therapy and/or vaccine. Several attempts have been undertaken to design such a vaccine and include the development of a life attenuated CHIKV strain and, more recently, a VLP-based approach [17]. VLPs have safety profiles very similar to those of subunits and immunogenic properties comparable to killed vaccines. It has been shown that there is great potential for the baculovirus expression system to generate effective (subunit) vaccines [21] and several vaccines produced in this system are on the market or in the process of registration. Furthermore, CHIKV is transmitted by mosquitoes and replicates to high viral titers in mosquitoes cells, thus it might be beneficial to express CHIKV proteins in insect cells [44]. In this research, vaccine candidates were generated consisting of individual CHIKV glycoproteins expressed in insect cells using recombinant baculoviruses.

CHIKV glycoprotein genes were cloned downstream of the polyhedrin promoter of baculovirus *Ac*MNPV to analyze individual expression of E1 and E2 with their native signal peptides and in the presence or absence of their respective C-terminal transmembrane domains. Results of SDS-PAGE and western blot analyses indicated that all proteins were expressed at high levels in *Sf*21 cells and that a substantial fraction of these proteins was processed in a similar fashion as during a natural CHIKV infection. The glycosylation patterns and resulting size changes found for CHIKV E1 and E2 appear to correspond with the postulated number of glycosylation sites in E1 and E2 (1 and 2, respectively). Our findings are in agreement with results obtained in glycoprotein expression studies for other alphaviruses and for the related *Rubellavirus*, that used recombinant baculoviruses [26,27,45,46].

We have shown that the glycoproteins travel through the ER, which is very sensitive to any homeostatic alterations and disturbances. Such ER stress is induced by protein misfolding, considerable protein overproduction and inhibition of N-linked glycosylation [47]. Generally, two baculovirus proteins cathepsin and chitinase are abundantly expressed and the latter tends to accumulate in the ER [48], thereby clogging up the ER and competing with recombinant proteins. Even though chitinase is deleted from the recombinant baculovirus backbone, it appears that the CHIKV glycoproteins are expressed in such massive amounts, that the unfolded protein response (UPR), which is normally induced in response to ER-stress [49], is not sufficient to mitigate ER stress, as concluded from the fact that a fraction of the 6KE1, 6KE1ΔTM, E3E2 and E3E2ΔTM appear in their unprocessed forms. Incomplete glycosylation and retention in the ER of intracellular viral glycoproteins expressed at very high levels in baculovirus-infected cells is common [50], but this did not compromise membrane localization and subunit secretion.

While glycosylation of recombinant CHIKV-E1 and -E2 was relatively efficient, expression analysis with the baculoviruses Ac-E3E2 and -E3E2ΔTM indicated that only a small fraction was cleaved by cellular furin. This, however, did not prevent secretion of the uncleaved, glycosylated E3E2 precursor as observed in the furin inhibition experiment and during metal-affinity chromatographay purification of secreted E2 subunits. We can therefore conclude that not furin activity, but rather the accumulation and retention of non-glycosylated E3E2 precursors in the ER, is limiting subunit secretion. This phenomenon has been well described for high level expression in insect cells of CSFV-E2, a similar glycoprotein from a different virus [50].

Although E3 plays a major role during CHIKV replication in the formation of E1 and E2 heterodimers [10], and the presence of uncleaved E3E2 in progeny alphavirus particles induces defects in virus production [51], it is not known if the presence of E3 has negative effects on the stability, functionality or antigenicity of E2 subunits. Considering that alphavirus virions with incorporated uncleaved E3E2 are able to bind efficiently to the cell surface, which is modulated by E2 [52], we expect that E3E2 is still sufficiently immunogenic. In fact, a recent report has shown that E3 also harbours protective epitopes [53]. Future studies might further explicate the effect of glycosylation on subunit antigenicity and epitope presentation.

Confocal microscopy showed that E1 and E2 are present at the surface of *Sf*21 cells. Since cells are able to secrete uncleaved, but glycosylated E3E2ΔTM, it is likely that cells expressing E3E2 display the protein in its glycosylated configuration on the surface of the cell. The reason that displayed or secreted proteins are all glycosylated, might be explained simply by the fact that glycosylation occurs prior to entering of the secretory pathway. Cells infected with Ac-6KE1 were able to form syncytia, indicating that E1 protein displayed at the surface was able to induce membrane fusion. Subjecting Ac-6KE1, Ac-E3E2 and Ac-GFP infected *Sf*9-ET cells to a range of acidified Sf900 II medium (pH = 5.0, 5.5, 5.8 and 6.4) excluded the possibility that syncytia formation was induced at pH 5.8 by the baculovirus fusion protein GP64, which is also expressed at the surface of infected cells [42]. The pH of Sf900 II medium was in the same range as it would be during endocytosis (pH5.5-pH5.8). Surprisingly, fusogenic activity was also shown at a pH = 6.4, which is expected to be slightly out of the fusogenic range of E1. This phenomenon was most likely caused by the addition of cholesterol to the culture medium. Cholesterol is known to be an important factor in alphavirus fusion [40], and a supplement activates CHIKV-E1 to be fusogenic. Whether its fusogenicity means that E1 is present in the form of homotrimers on the surface of the cell needs experimental confirmation.

The virus neutralization test shows that the rabbit polyclonal antiserum elicited against purified CHIKV-E2ΔTM is able to neutralize CHIKV. This is the first time that a secreted form of CHIKV-E2, expressed by the recombinant baculovirus-insect cell expression system elicits neutralizing antibodies in rabbits. No neutralizing antibodies were detected in the serum of E1ΔTM vaccinated rabbits, which is not entirely unexpected since E1 is partially covered by E2 in mature virions [54]. This is clear proof for antigenicity of the E2 subunit vaccine candidate, which will now be further studied in vaccination trials in an animal model. The fact that expression of CHIKV-glycoproteins in insect cells results in correct processing similar to the processing found during wildtype infections, proposes it to be a very useful and promising expression system in the generation and development of alphavirus subunit vaccines. In addition, proteins appear to retain their original function, paving the way for recombinant baculoviruses to be used in functionality studies in insect cells.

## Conclusions

In conclusion, this study has shown that expression of full-length CHIKV 6KE1 and E3E2 glycoproteins using baculovirus vectors in insect cells leads to glycosylation, furin processing, plasma membrane translocation of E1 and E2, and CHIKV-E1 retains its functional activity as a membrane fusion protein. In addition, the deletion of the C-terminal transmembrane domain enables secretion of glycoprotein E1 and E2 independent of furin cleavage. The purified, glycosylated and secreted CHIKV E2 subunit induced neutralizing antibodies in rabbits and can now be tested for its ability to provide protection against CHIKV challenge in an animal model.

## Methods

### Cells and viruses

*Spodoptera frugiperda *(*Sf*21) insect cells (Invitrogen) were maintained in tissue culture flasks (Greiner) as a monolayer culture in Grace's insect medium (Invitrogen), supplemented with 10% fetal bovine serum (FBS, Hyclone). *Sf*9-easy titer (ET) cells [41] were maintained as a monolayer culture in Sf900 II (Invitrogen) serum free medium. The Bac-to-Bac baculovirus expression system (Invitrogen) based upon *Autographa californica *multicapsid nucleopolyhedrovirus (AcMNPV) was used to generate recombinant baculoviruses. The bacmid backbone AcΔcc contained a deletion of the promoters and large parts of the ORFs of cathepsin and chitinase, thereby leaving flanking essential genes intact [55]. Furthermore, the p10 promoter and ORF were deleted from the AcΔcc backbone by replacing these elements with a zeocin resistance marker by Lambda Red recombination [56,57]. This zeocin resistance marker was flanked by modified loxP sequences as described by [58] for bacterial genome modifications. This allowed the subsequent removal of the marker gene with Cre recombinase.

Baculovirus titers were determined by end point dilution assay and expressed as tissue culture infectious dose 50 (TCID_50_) per ml. Viral RNA of Chikungunya virus isolate S27 (Tanzania, 1953) provided by Erasmus Medical Center (EMC), Rotterdam, was used as a source for cDNA synthesis.

CHIKV-S27 virus stocks were produced in BHK-21 cells in a BSL3 laboratory. Input virus was removed by extensive washing, and supernatant containing infectious virus was harvested four days post infection (pi) and filtered. Virus titration assays were performed on BHK-21 cell monolayers in 96 wells plate and titer was calculated using the Karber method. Virus stock titers were expressed as TCID_50 _per ml. CHIKV stocks containing 10^7 ^TCID_50 _/ml were aliquoted and stored at -80°C.

### Construction of recombinant baculoviruses encoding CHIKV glycoproteins

Amplicons of CHIKV 6KE1 and E3E2 were generated using SuperScript One-Step RT-PCR with Platinum Taq (Invitrogen), cloned in pGEM-Teasy vectors (Promega) and sequenced (Eurofins Operon, Germany). CHIKV cDNA sequences encoding 6KE1, 6KE1ΔTM, E3E2 and E3E2ΔTM (Figure 1) were PCR amplified from these plasmids using extended primers (6K-F; ggggacaagtttgtacaaaaaagcaggcttaggatccaccatggccacataccaagaggctgc, E3-F; ggggacaagtttgtacaaaaaagcaggcttaggatccaccatgagtcttgccatcccagttatg, E1-R; ggggaccactttgtacaagaaagctgggtaaagcttctaatgatgatgatgatgatgcatgtgcctgctgaacgacacgc, E1ΔTM-R; ggggaccactttgtacaagaaagctgggtaaagcttctaatgatgatgatgatgatgcatccatgacatcgccgtagcgg, E2-R; ggggaccactttgtacaagaaagctgggtaaagcttctaatgatgatgatgatgatgctgcagcgctttagctgttctgatgc and E2ΔTM-R; ggggaccactttgtacaagaaagctgggtaaagcttctaatgatgatgatgatgatgctgcagcagctcataataatacagaa) that introduce AttB recombination sites (underlined) to enable Gateway^® ^cloning (Invitrogen). Resulting PCR products were cloned into pDONR207 donor plasmid (Invitrogen) and subsequently transferred to the pFastBac1 derivative pDEST8 (Invitrogen). Recombinant baculoviruses encoding the CHIKV protein constructs were generated using the Bac-to-Bac baculovirus expression system (Invitrogen), resulting in Ac-6KE1, Ac-6KE1ΔTM, Ac-E3E2 and Ac-E3E2ΔTM.

### Protein analysis

For analysis of protein expression, 6 × 10^6 ^*Sf*21 cells were seeded into 75 cm^2 ^culture flasks. Cells were infected with recombinant virus at a multiplicity of infection (MOI) of 10 TCID_50 _units per cell. Cells were harvested 72 hpi and washed twice in 1 ml phosphate buffered saline (PBS). Finally, cells were resuspended in 500μl PBS and stored at -20°C. Whole cell lysates were analyzed by sodium dodecyl sulphate polyacrylamide gel electrophoresis (SDS-PAGE) and Coomassie brilliant blue staining. A lysate of CHIKV-infected Ap61 mosquito cells was used as positive control. Proteins were transferred to an immobilon transfer membrane (Millipore) for Western blot (WB) analyses. Membranes were blocked in PBS + 0.1% Tween-60 (PBST) containing 3% skim milk for 1 h at 37°C. Next, the membranes were washed with PBST and incubated with rabbit polyclonal antiserum (supplied by Nobilon International BV) at a dilution of 1:15,000 for 1 h at 37°C. After incubation, membranes were washed and treated with 1:3,000 diluted Alkaline Phosphatase (AP) conjugated goat anti-rabbit IgG antibodies (Sigma) for 45 min at 37°C. Proteins were detected by NBT/BCIP staining (Roche).

### Tunicamycin assay

A tunicamycin assay was performed to analyze the glycosylation status of recombinant CHIKV E1 and E2. *Sf*21 cells were infected with recombinant baculoviruses at an MOI of 10 TCID_50 _units per cell. Cells were incubated at 27°C in the presence of 10 μg/ml tunicamycin (Sigma) and harvested 72 hpi. Protein glycosylation was visualized using Western blotting and Periodic acid Schiff staining. Proteins were blotted on an immobilon transfer membrane (Millipore) and soaked in PAS solution (1% perjodium acid in 3% acetic acid) for 15 min at room temperature (RT). The membrane was washed several times in water and was incubated in the dark for 15 minutes at RT with Schiff's reagent. Finally, the membrane was washed for 5 min in 50% Na_2_CO_3_.

### Furin inhibition assay

*Sf*21 cells were infected with Ac-E3E2 and E3E2ΔTM at an MOI of 10 in Grace's insect medium (Invitrogen) without FBS. Cells were washed twice with medium and incubated with medium containing 50 μM of Furin Inhibitor I (Calbiochem). Cells were harvested at 72 hpi and the medium fraction was separated from the cell fraction by centrifugation. Protein processing was analyzed by Western blotting.

### Immunostaining of baculovirus infected *Sf*21 cells

*Sf*21 cells were infected with Ac-E3E2 and Ac-6KE1 at a MOI of 10 to determine surface expression of CHIKV-E1 and -E2. Cells were harvested 48 hpi and washed with PBS. Next, the cells were incubated for 1 h at RT with PBS+5%FBS containing 1:5,000 diluted rabbit α-CHIKV-E1 and rabbit α-CHIKV-E2 polyclonal antibodies. Cells were washed 3 times and incubated for 1 h at RT with PBS+5%FBS containing 1:1,000 diluted goat-anti-rabbit-Alexa Fluor 488 (Invitrogen). Cells were analyzed using laser scanning confocal microscopy on a Zeiss LSM 510 Meta, Axiovert 100 m (Zeiss) with Argon laser (488 nm) and images were analyzed with Zeiss LSM image browser.

### Syncytium formation assay

The syncytium formation assay was performed by infecting *Sf*9-easy titer (ET) cells [41] with Ac-E3E2,Ac-6KE1 at an MOI of 10 in Sf900 II medium (pH = 6.4 ), supplemented with cholesterol (0.2 mg/ml, Sigma). A recombinant AcMNPV expressing GFP (Ac-GFP) was used as a negative control. Syncytium formation was induced 72 hpi, by subjecting infected cells for 2 minutes to acidified medium with pH = 5.8, pH = 5.5 and pH = 5.0. Syncytium formation was scored 4 h post induction using fluorescence microscopy.

### Purification of secreted CHIKV-E1 and -E2 subunits from insect cells

*Sf*21 cells were infected with Ac-6KE1ΔTM and Ac-E3E2ΔTM at an MOI of 10 in Grace's insect medium without FBS and were incubated for 72 h at 27°C. The recombinant proteins were purified from the medium fraction using Talon^® ^spin columns (Clontech) according to the manufacturer's protocol. Bound protein fractions were eluted with 150 mM imidazol and total protein content was determined via a Bradford protein assay (Biorad) according to the manufacturer's procedure.

### Virus neutralization test

Virus neutralizing antibody titers in sera of rabbits vaccinated with purified CHIKV-E1ΔTM and E2ΔTM, were determined as follows. Serial two-fold dilutions of heat-inactivated rabbit sera were prepared in triplicate in 96-wells plate and 100 TCID_50 _of CHIKV suspension was added to each well. After one hour of incubation at 37°C, 1 × 10^4 ^BHK-21 cells were added to each well and plates were incubated for another four days. Neutralizing titers were determined microscopically and expressed as the reciprocal of the highest serum dilution still giving 100% suppression of cytopathic effect.

## Abbreviations

AP: Alkaline Phosphatase; C: Capsid; CBB: Coomassie Brilliant Blue; CHIKV: Chikungunya Virus; ER: Endoplasmic Reticulum; FBS: Fetal Bovine Serum; GFP: Green Fluorescent Protein; HBM: Honey Bee Melittin; Mabs: Monoclonal antibodies; MOI: Multiplicity of Infection; ORF: Open Reading Frame; PAS: Periodic Acid Schiff; PBS: Phosphate Buffered Saline; PE2: Precursor E2 (E3E2); *Sf*21: *Spodoptera frugiperda *21; SINV: Sindbis Virus; TCID_50_: Tissue Culture Infective Dose 50%; VLP: Virus-Like Particle.

## Competing interests

The author declares that they have no competing interests.

## Authors' contributions

SM carried out the expression and secretion studies, glycosylation and furin inhibiton assays, immunofluorescence and syncytia formation assays and drafted the manuscript. CG carried out the subunit purification and participated in creating recombinant baculoviruses. BM carried out the virus neutralization test. PA participated in creating recombinant baculoviruses. JH participated in antibody generation. MO participated in creating recombinant baculoviruses. RG participated in the design and coordination of the study. JV participated in the design of the study and helped to draft the manuscript. GP conceived of, designed and coordinated the study and helped to draft the manuscript. All authors read and approved the final manuscript.
